# Advance Care Planning (ACP) in Medicare Beneficiaries with Heart Failure

**DOI:** 10.1007/s11606-024-08604-1

**Published:** 2024-05-20

**Authors:** Seuli Bose Brill, Sean R. Riley, Laura Prater, Patrick M. Schnell, Anne L. R. Schuster, Sakima A. Smith, Beth Foreman, Wendy Yi Xu, Jillian Gustin, Yiting Li, Chen Zhao, Todd Barrett, J. Madison Hyer

**Affiliations:** 1 Division of General Internal Medicine, Department of Internal Medicine, The Ohio State University College of Medicine, 2050 Kenny Road, Columbus, OH 43215 USA; 2 Center for Health Outcomes in Medicine Scholarship and Service, Department of Internal Medicine, The Ohio State University College of Medicine, Columbus, OH USA; 3 Division of Health Services Management and Policy, The Ohio State University College of Public Health, Columbus, OH USA; 4 Division of Biostatistics, The Ohio State University College of Public Health, Columbus, OH USA; 5 Department of Biomedical Informatics, The Ohio State University College of Medicine, Columbus, OH USA; 6 Division of Cardiology, The Ohio State University College of Medicine, Columbus, OH USA; 7 Division of Palliative Medicine, Department of Internal Medicine, The Ohio State University College of Medicine, Columbus, OH USA; 8 University of Miami, Miller School of Medicine, Miami, FL USA; 9 Ohio State University Ross Heart Hospital, Columbus, OH USA; 10 Center for Biostatistics, The Ohio State University Wexner Medical Center, Columbus, OH USA

**Keywords:** end-of-life care, advance care planning, heart failure, healthcare utilization, healthcare expenditures

## Abstract

**Background:**

Heart failure is a leading cause of death in the USA, contributing to high expenditures near the end of life. Evidence remains lacking on whether billed advance care planning changes patterns of end-of-life healthcare utilization among patients with heart failure. Large-scale claims evaluation assessing billed advance care planning and end-of-life hospitalizations among patients with heart failure can fill evidence gaps to inform health policy and clinical practice.

**Objective:**

Assess the association between billed advance care planning delivered and Medicare beneficiaries with heart failure upon the type and quantity of healthcare utilization in the last 30 days of life.

**Design:**

This retrospective cross-sectional cohort study used Medicare fee-for-service claims from 2016 to 2020.

**Participants:**

A total of 48,466 deceased patients diagnosed with heart failure on Medicare.

**Main Measures:**

Billed advance care planning services between the last 12 months and last 30 days of life will serve as the exposure. The outcomes are end-of-life healthcare utilization and total expenditure in inpatient, outpatient, hospice, skilled nursing facility, and home healthcare services.

**Key Results:**

In the final cohort of 48,466 patients (median [IQR] age, 83 [76–89] years; 24,838 [51.2%] women; median [IQR] Charlson Comorbidity Index score, 4 [2–5]), 4406 patients had an advance care planning encounter. Total end-of-life expenditure among patients with billed advance care planning encounters was 19% lower (95% CI, 0.77–0.84) compared to patients without. Patients with billed advance care planning encounters had 2.65 times higher odds (95% CI, 2.47–2.83) of end-of-life outpatient utilization with a 33% higher expected total outpatient expenditure (95% CI, 1.24–1.42) compared with patients without a billed advance care planning encounter.

**Conclusions:**

Billed advance care planning delivery to individuals with heart failure occurs infrequently. Prioritizing billed advance care planning delivery to these individuals may reduce total end-of-life expenditures and end-of-life inpatient expenditures through promoting use of outpatient end-of-life services, including home healthcare and hospice.

**Supplementary Information:**

The online version contains supplementary material available at 10.1007/s11606-024-08604-1.

## INTRODUCTION

Since the advent of advance care planning (ACP) billing codes in 2016, policies increasingly tie their use to healthcare quality measures in value-based payment models.^1,2^ Since the Centers for Medicare and Medicaid Services (CMS) established billing mechanisms for ACP services to the Physician Fee Schedule under Current Procedural Terminology (CPT) codes 99,497 and 99,498, ACP billing has increased each year preferences for future medical care in preparation for severe illness affecting the ability to communicate or make decisions.^3–5^ These ACP billing codes reimburse for time-based physician or advance practice provider-led, multi-disciplinary ACP communication involving the patient, family members, and/or surrogate decision makers. CPT code 99,497, the bas ACP code, requires 16–30 min of time spent on ACP. The CPT code 99,498 is an add-on code for billing each additional 30 min of ACP communication. ACP billing codes require clinicians to include specific documentation elements about the discussion within the associated clinical encounter, including individuals present, voluntary nature, details about completed or available advance directives, medical necessity, and face-to-face time spent.^6^ While ACP discussions with patients with complex or life-limiting illness may have more imminent applicability to their medical care plans, current ACP billing patterns demonstrate that Medicare beneficiaries with high disease burden receive billed ACP services less frequently than their healthier counterparts.^1,7^

While ACP consistently demonstrates reduction in end-of-life (EOL) intensive care among persons with serious illness,^8–10^ the impact of ACP on EOL healthcare utilization, including hospice enrollment patterns, remains unclear.^11,12^ Inconsistent findings about the impacts of ACP billing may lie in attempts to juxtapose studies focused on varying populations, ACP timing, and outcomes. These mixed findings, combined with parallel inconsistencies within the decades-long trajectory of ACP research, have spurred debate about policies focused on increasing ACP uptake.^13–17^ ACP advocates point to studies demonstrating the role of ACP in promoting clinician and surrogate decision maker alignment with patient preferences, increasing patient and caregiver satisfaction, and decreasing surrogate decision maker distress.^13,18^ ACP skeptics point to failures, such as stymied clinical translation and inconsistent evidence of its utility, including impact on patient quality-of-life.^16^ Lack of consensus about the potential benefits of ACP remains coupled with, and exacerbated by, persistently low delivery of these services nationally.^19,20^

These ACP challenges have downstream implications in diseases of high public health significance, such as heart failure (HF).^21^ HF currently affects approximately 6.2 million American adults and is expected to affect 8 million American adults by 2030.^22,23^ HF remains a leading cause of hospitalization and readmissions among Medicare beneficiaries.^24^ Despite advances in medical therapy, the overall 1-year HF mortality rate among Medicare beneficiaries approaches 30%.^22^ The prevalence, morbidity, and mortality of HF are even higher among racial minorities.^25^ The age-adjusted death rate of HF is highest among Black men (118.2 per 100,000). ^26^ Disparities in HF clinical outcomes are compounded by disparities in ACP delivery; Black and Hispanic patients are less likely than White patients to have had an ACP conversation.^27,28^

ACP may provide benefits, such as improved quality of life and reduction in depressive symptoms, to individuals living with HF, but many gaps in knowledge persist, including optimal timing and frequency of ACP delivery. ^29–31^ Despite American Heart Association (AHA) guidance delineating ACP as a critical component of HF care management, persons with HF continue to receive billed ACP services infrequently.^32–36^ This quantitative study aims to determine whether billed ACP services delivered to HF-diagnosed Medicare beneficiaries within the last 12 months of life are associated with changes in the type and quantity of healthcare utilization in the last 30 days of life.

## METHODS

### Data Source

Data were derived from 2016 to 2020 100% Medicare Standard Analytical Files (SAFs). The SAFs were developed from fee-for-service claims and are maintained by CMS. They contain encounter-level data including but not limited to date of encounter, expenditures, and diagnosis and procedure codes. They also include patient-level data such as age, sex, race/ethnicity, state and county residence, and date of death. For this study, patients were included if they were enrolled in Medicare Parts A and B, aged 65 years or older, died during the study period, and had a diagnosis of HF. Diagnosis codes were taken from the Charlson Comorbidity Index (CCI) (eTable 1).^37,38^ This study was exempt from review by the local institutional review board per institutional policy.

### Study Variables

**Figure 1:**
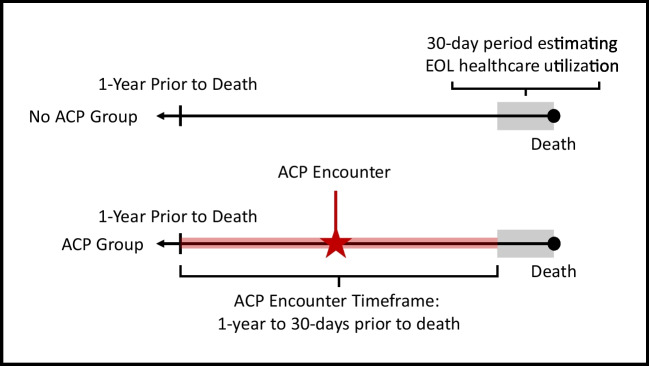
Timeframe for identification of billed ACP exposure prior to death.

The independent variable of this study was ACP utilization. In alignment with prior ACP literature, we assessed ACP utilization between 1 year and 30 days before death (Fig. 1).^11^ Patients who received ACP services were identified using CPT codes 99,497 and 99,498. Patients without an ACP encounter were considered controls and propensity score matched to patients with an ACP encounter at a 10:1 ratio on age of death, CCI, and with exact matching on sex, race, region, and year of death. Patient’s region of residence was categorized according to the US Census Bureau.

The outcomes were various measures of EOL healthcare utilization, including total expenditure, as well as five categories of healthcare: inpatient, outpatient, hospice, skilled nursing facility (SNF), and home healthcare (HHC) utilization during the last 30 days of life. For each category, any utilization was recorded as a yes/no and among those who had utilization, the amount of expenditure associated with the utilization. In addition, the number of encounters/admissions and the length of stay were also evaluated.

### Statistical Analysis

Descriptive statistics were calculated as median (25th–75th percentiles [IQR]) and frequency (relative frequency [%]) for continuous and categorical measures, respectively. To assess the effect of ACP encounters on EOL healthcare utilization, a range of modeling techniques were utilized. For presence of utilization (yes/no), multivariable logistic regression was utilized to produce odds ratios (OR) and their 95% confidence intervals (95% CIs). For number of encounters/admissions and lengths of stay, multivariable negative binomial regression with a log link was utilized. For expenditure outcomes, multivariable gamma regression with a log link was utilized. For negative binomial and gamma regression models, incidence rate ratios (IRR) and their 95% CIs were estimated. All analyses were performed with an alpha level of 0.05 using SAS v9.4.

Two sets of sensitivity analyses were performed to clarify the effect of ACP on EOL healthcare utilization: analyses stratified by EOL outpatient engagement (i.e., at least one outpatient encounter within the 30-day period before death) and expanding the classification of patients with ACP encounters to delineate between patients with only one ACP encounter and those with multiple ACP encounters.

## RESULTS

### Population Characteristics

A total of 48,466 patients with HF were included in this study (Table 1). Within this sample, 4406 patients had an ACP encounter and 44,060 patients did not have an ACP encounter prior to study end. Approximately half of the cohort were female (51.2%; *n* = 24,838), 88.1% were white (*n* = 42,680), the median age was 83 years (IQR, 76–89), and the median CCI was 4 (IQR, 2–5). Given that this cohort is the result of a 10:1 PSM analysis, balance was achieved on all patient factors, including age, race, gender, age, CCI, region of residence, and year of death. Among those patients with at least one ACP encounter, 8.8% (*n* = 388) had two or more ACP encounters. On overall expenditure, median EOL expenditures were more than $2000 higher among patients with no ACP encounter (ACP, $9890 vs. no ACP, $11,930).

**Table 1:** Descriptive Statistics are Presented as Median (IQR) and Frequency (%) for Continuous and Categorical Measures, in Total and Stratified by Billed ACP Encounter Status ^46,47^

	Total	No ACP *N* = 44,060	ACP *N* = 4406
Number of ACP encounters			
1	4018 (91.2%)	-	4018 (91.2%)
2	333 (7.6%)	-	333 (7.6%)
3 +	55 (1.3%)	-	55 (1.3%)
Age	83 (76, 89)	83 (76, 89)	83 (76, 89)
Charlson Comorbidity Index	4 (2, 5)	4 (2, 5)	4 (2, 5)
Female	24,838 (51.2%)	22,580 (51.2%)	2258 (51.2%)
Race			
White	42,680 (88.1%)	38,800 (88.1%)	3880 (88.1%)
Black	3586 (7.4%)	3260 (7.4%)	326 (7.4%)
Other	2200 (4.5%)	2000 (4.5%)	200 (4.5%)
Region			
Midwest	7898 (16.3%)	7180 (16.3%)	718 (16.3%)
Northeast	12,287 (25.4%)	11,170 (25.4%)	1117 (25.4%)
South	15,378 (31.7%)	13,980 (31.7%)	1398 (31.7%)
West	12,903 (26.6%)	11,730 (26.6%)	1173 (26.6%)
Year of death			
2016	1573 (3.2%)	1430 (3.2%)	143 (3.2%)
2017	5379 (11.1%)	4890 (11.1%)	489 (11.1%)
2018	10,098 (20.8%)	9180 (20.8%)	918 (20.8%)
2019	14,201 (29.3%)	12,910 (29.3%)	1291 (29.3%)
2020	17,215 (35.5%)	15,650 (35.5%)	1565 (35.5%)
	30-day outcomes		
Total expenditure ($)	11,706 (5746, 23,668)	11,925 (5806, 24,136)	9883 (5181, 19,357)
Outpatient			
Any utilization	23,394 (48.3%)	20,345 (46.2%)	3049 (69.2%)
# of encounter	0 (0, 2)	0 (0, 2)	2 (0, 4)
Total expenditure ($)	0 (0, 659)	0 (0, 618)	198 (0, 1089)
Inpatient			
Any utilization	27,511 (56.8%)	25,363 (57.6%)	2148 (48.8%)
# of admissions	1 (0, 1)	1 (0, 1)	0 (0, 1)
Total length of stay (days)	2 (0, 8)	2 (0, 9)	0 (0, 6)
Total expenditure ($)	6960 (0, 16,612)	7300 (0, 17,089)	0 (0, 12,805)
Hospice			
Any utilization	24,370 (50.3%)	21,897 (49.7%)	2473 (56.1%)
Total length of stay (days)	0 (0, 12)	0 (0, 12)	2 (0, 17)
Total expenditure ($)	0 (0, 3490)	0 (0, 3421)	825 (0, 4117)
Skilled nursing facility			
Any utilization	10,228 (21.1%)	9255 (21.0%)	973 (22.1%)
Total length of stay (days)	0 (0, 0)	0 (0, 0)	0 (0, 0)
Total expenditure ($)	0 (0, 0)	0 (0, 0)	0 (0, 0)
Home healthcare			
Any utilization	6848 (14.1%)	6200 (14.1%)	648 (14.7%)
Total length of stay (days)	0 (0, 0)	0 (0, 0)	0 (0, 0)
Total expenditure ($)	0 (0, 0)	0 (0, 0)	0 (0, 0)

**Abbreviations:** *IQR*, interquartile range; *ACP*, advance care planning

### ACP vs. No ACP Encounters

EOL healthcare utilization differed considerably between patients that did and patients that did not have an ACP encounter (Table 1). Outpatient and hospice utilization rates were higher among patients with an ACP encounter: where, 69.2% and 56.1% of patients with an ACP encounter had outpatient and hospice utilization at the EOL, respectively, compared with 46.2% and 49.7% for patients with no ACP encounter. Conversely, patients with an ACP encounter had lower rates of inpatient utilization (ACP, 48.8% vs. no ACP, 57.6%).

Overall EOL expenditure totals were 19% lower (95% CI, 0.77–0.84) among patients with an ACP encounter compared with patients without an ACP encounter (Table 2). Regarding outpatient utilization, patients with ACP encounter had 2.65 times higher odds (95% CI, 2.47–2.83) of having outpatient utilization with an expected total outpatient expenditure 33% higher (95% CI, 1.24–1.42) compared with patients without an ACP encounter. Dissimilarly, ACP encounter patients had 34% lower odds (95% CI, 0.61–0.70) of having an EOL inpatient admission and 31% lower inpatient expenditures (95% CI, 0.64–0.74) compared with patients with no ACP encounter.

**Table 2:** Multivariable Analysis Results are Presented as Incidence Rate Ratio (IRR) or Odds Ratio (OR) as well as the Corresponding 95% Confidence Intervals for all Utilization Outcomes, Comparing ACP Encounter with no ACP Encounter

	*IRR/^£^OR	95% CI	*p*
Total expenditure ($)	*0.81	0.77–0.84	< 0.001
Outpatient			
Any utilization	^£^2.65	2.47–2.83	< 0.001
# of encounter	*2.34	2.24–2.44	< 0.001
Total expenditure ($)	*1.33	1.24–1.42	< 0.001
Inpatient			
Any utilization	^£^0.66	0.61–0.70	< 0.001
# of admissions	*0.84	0.81–0.87	< 0.001
Total length of stay (days)	*0.68	0.64–0.71	< 0.001
Total expenditure ($)	*0.69	0.64–0.74	< 0.001
Hospice			
Any utilization	^£^1.32	1.24–1.41	< 0.001
Total length of stay (days)	*1.26	1.17–1.36	< 0.001
Total expenditure ($)	*1.18	1.10–1.27	< 0.001
Skilled nursing facility			
Any utilization	^£^1.07	0.99–1.16	0.07
Total length of stay (days)	*1.12	0.98–1.27	0.10
Total expenditure ($)	*1.14	1.05–1.24	0.002
Home healthcare			
Any utilization	^£^1.06	0.97–1.16	0.22
Total length of stay (days)	*1.03	0.89–1.20	0.68
Total expenditure ($)	*0.96	0.89–1.03	0.24

**Abbreviations:** *IRR*, incidence rate ratio; *OR*, odds ratio; *ACP*, advance care planning

Overall EOL expenditures as well as outpatient and inpatient utilization were similar among African-American and White patients (Table 3). Among African-American patients with an ACP encounter, overall expenditures were 16% lower (95% CI, 0.72–0.98) compared with African-American patients without an ACP encounter, while among White patients with an ACP encounter, overall expenditures were 19% lower (95% CI, 0.77–0.84) compared to white patients without an ACP encounter. African-American patients with an ACP had 2.63 higher odds (95% CI, 2.06–3.37) of outpatient utilization and 30% lower odds (95% CI, 0.54–0.89) of inpatient utilization, while White patients with an ACP encounter had 2.65 higher odds (95% CI, 2.47–2.85) of outpatient utilization and 35% lower odds (95% CI, 0.60–0.70) of inpatient utilization.

**Table 3:** Multivariable Analysis Results are Presented as Incidence Rate Ratio (IRR) or Odds Ratio (OR) as well as the Corresponding 95% Confidence Intervals for all Utilization Outcomes, Comparing ACP Utilization Between White and Black/African-American Patients

	White patients			Black/African-American patients		
	*IRR/^£^OR	95% CI	*p*	*IRR/^£^OR	95% CI	*p*
Total expenditure ($)	*0.81	0.77–0.84	< 0.001	*0.84	0.72–0.98	< 0.001
Outpatient						
Any utilization	^£^2.65	2.47–2.85	< 0.001	^£^2.63	2.06–3.37	< 0.001
# of encounter	*2.37	2.26–2.48	< 0.001	*2.21	1.88–2.58	< 0.001
Total expenditure ($)	*1.34	1.25–1.44	< 0.001	1.27	0.99–1.63	0.059
Inpatient						
Any utilization	^£^0.65	0.60–0.70	< 0.001	^£^0.70	0.54–0.89	< 0.001
# of admissions	*0.84	0.80–0.88	< 0.001	*0.89	0.78–1.02	0.09
Total length of stay (days)	*0.67	0.63–0.71	< 0.001	*0.75	0.61–0.92	0.006
Total expenditure ($)	*0.68	0.63–0.74	< 0.001	*0.80	0.61–1.04	0.09
Hospice						
Any utilization	^£^1.31	1.22–1.40	< 0.001	^£^1.20	0.95–1.52	0.13
Total length of stay (days)	*1.22	1.13–1.32	< 0.001	*1.40	1.08–1.82	0.011
Total expenditure ($)	*1.14	1.06–1.23	0.001	*1.33	1.03–1.72	0.026
Skilled nursing facility						
Any utilization	^£^1.10	1.01–1.19	0.026	^£^1.02	0.77–1.36	0.87
Total length of stay (days)	*1.13	0.98–1.30	0.10	*1.14	0.70–1.85	0.59
Total expenditure ($)	*1.16	1.06–1.27	0.001	*1.10	0.81–1.50	0.54
Home healthcare						
Any utilization	^£^1.06	0.96–1.17	0.25	^£^0.99	0.70–1.39	0.94
Total length of stay (days)	*1.04	0.89–1.22	0.65	*0.82	0.48–1.40	0.47
Total expenditure ($)	*0.96	0.89–1.04	0.36	*0.83	0.63–1.09	0.19

**Abbreviations:** *IRR*, incidence rate ratio; *OR*, odds ratio; *ACP*, advance care planning

### No ACP vs. 1 ACP vs. Multiple ACP Encounters

The effect of ACP was magnified in cases where a patient had multiple ACP encounters compared with patients who only had one ACP encounter (Table 4). Of note, patients with only one ACP encounter had 18% lower total EOL expenditure (95% CI, 0.78–0.88) whereas patients with multiple ACP encounters had 31% lower total EOL expenditure (95% CI, 0.60–0.79) compared with patients who never had an ACP encounter. Similarly, patients with multiple ACP encounters had lower odds of inpatient utilization (multiple ACP, 0.41 vs. 1 ACP, 0.69), shorter lengths of stay (multiple ACP, 0.47 vs. 1 ACP, 0.70), and less total inpatient expenditure (multiple ACP, 0.50 vs. 1 ACP, 0.71).

**Table 4:** Multivariable Analysis Results are Presented as Incidence Rate Ratio (IRR) or Odds Ratio (OR) as well as the Corresponding 95% Confidence Intervals for all Utilization Outcomes, Comparing one ACP Encounter or Multiple ACP Encounters with no ACP Encounter

	1 ACP enc. vs. no ACP enc			Multiple ACP encs. vs. no ACP enc		
	*IRR/^£^OR	95% CI	*p*	*IRR/^£^OR	95% CI	*p*
Total expenditure ($)	*0.82	0.78–0.88	< 0.001	*0.69	0.60–0.79	< 0.001
Outpatient						
Any utilization	^£^2.63	2.45–2.82	< 0.001	^£^2.82	2.26–3.52	< 0.001
# of encounter	*2.32	2.21–2.42	< 0.001	*2.53	2.21–2.90	< 0.001
Total expenditure ($)	*1.32	1.23–1.42	< 0.001	*1.38	1.11–1.72	< 0.001
Inpatient						
Any utilization	^£^0.69	0.64–0.74	< 0.001	^£^0.41	0.33–0.52	< 0.001
# of admissions	*0.85	0.82–0.89	< 0.001	*0.69	0.61–0.79	< 0.001
Total length of stay (days)	*0.70	0.66–0.74	< 0.001	*0.47	0.39–0.56	< 0.001
Total expenditure ($)	*0.71	0.65–0.76	< 0.001	*0.50	0.40–0.63	< 0.001
Hospice						
Any utilization	^£^1.29	1.21–1.38	< 0.001	^£^1.71	1.39–2.11	< 0.001
Total length of stay (days)	*1.23	1.14–1.32	< 0.001	*1.61	1.28–2.02	< 0.001
Total expenditure ($)	*1.15	1.01–1.23	< 0.001	*1.54	1.23–1.92	< 0.001
Skilled nursing facility						
Any utilization	^£^1.09	1.01–1.18	0.030	^£^0.88	0.68–1.14	0.13
Total length of stay (days)	*1.13	0.98–1.30	0.08	*0.97	0.63–1.48	< 0.001
Total expenditure ($)	*1.16	1.06–1.27	< 0.001	*0.96	0.73–1.26	0.75
Home healthcare						
Any utilization	^£^1.03	0.93–1.13	0.59	^£^1.39	1.06–1.82	0.016
Total length of stay (days)	*0.99	0.85–1.16	0.91	*1.46	0.91–2.34	0.12
Total expenditure ($)	*0.94	0.87–1.01	0.10	*1.16	0.91–1.48	0.22

**Abbreviations:** *IRR*, incidence rate ratio; *OR*, odds ratio; *ACP*, advance care planning

### End-of-Life Outpatient Engagement

All patient characteristics were well balanced between patients who did and did not have an ACP encounter, irrespective of whether the patient had EOL outpatient engagement (eTables 2 & 3 in the Supplement). An ACP encounter was associated with 19% lower total EOL expenditures (95% CI, 0.78–0.85) among patients with EOL outpatient engagement and 25% lower total EOL expenditures (95% CI, 0.69–0.82) among patients without EOL outpatient engagement (eTable 4 in the Supplement). Diverging from the results of the overall study population, among patients with EOL outpatient engagement, having ACP encounter was associated with 12% decreased outpatient expenditure (95% CI, 0.84–0.92). While the directions of association between ACP encounters as well as inpatient and hospice utilization were similar with the overall cohort, more pronounced effects were observed in the cohort of patients without EOL outpatient engagement. More specifically, an ACP encounter was associated with 45% lower inpatient expenditures (95% CI, 0.48–0.63) among patients without EOL outpatient engagement compared with 28% lower expenditures (95% CI, 0.67–0.78) among patients with EOL outpatient engagement. With regard to any hospice utilization, an ACP encounter was associated with 2.32 times higher odds of utilizing hospice (95% CI, 2.04–2.63) among patients without EOL outpatient engagement compared with 1.36 times higher odds (95% CI, 1.25–1.47) among patients with EOL outpatient engagement.

## DISCUSSION

This study finds that individuals with HF who received billed ACP had 25% lower EOL healthcare expenditures compared with propensity score matched controls who did not receive billed ACP. However, this reduction in healthcare expenditure occurred through lower inpatient admission rates, likely substituted by increased outpatient healthcare service utilization, including HHC and hospice. Our findings suggest that the ACP-correlated reduction in healthcare expenditures among HF patients partially results from a shift in where healthcare services are delivered, rather than a reduction of EOL healthcare services. While our data source did not allow exploration of whether this shift reflected alignment with patient goals, recent studies have demonstrated that patients with HF who receive palliative communication overwhelmingly prefer home-based and outpatient EOL care over inpatient care.^39^

Our findings also demonstrate that persons with HF infrequently receive ACP, despite incremental increases in billed ACP since its inception in 2016, corroborating existing data on ACP trends.^19,20^ As most individuals with HF nearing the EOL have not received any billed ACP services, our findings suggest a significant opportunity to increase delivery of high value care through the incorporation of systematic billable ACP encounters into comprehensive HF care. Modifying payment policies to prioritize ACP delivery to individuals with HF may incentivize behaviors and processes necessary to increase ACP frequency. Such ACP strategies would not only align with AHA guidance concerning HF care, but may also result in reducing EOL expenditures through promoting outpatient EOL support over hospital-based care. Whichever way they are implemented, HF-focused ACP strategies must also address disparities in ACP delivery to ensure equitable downstream impacts. While our findings demonstrate that billed ACP encounters have a positive impact on outcomes for White and Black patients, our findings demonstrate that Black individuals with HF near the EOL are underrepresented in receipt of billed ACP encounters relative to the proportion of Black Medicare beneficiaries.

Our findings also suggest that iterative billed ACP, a pattern aligning with Institute of Medicine recommendations, ^40^ was associated with reduced total EOL healthcare expenditures more than a single billed ACP encounter. In addition, progressive increases in outpatient healthcare utilization accompanied reduction in total healthcare utilization among individuals who received 2 billed ACP encounters and 3 + billed ACP encounters respectively.

This study revealed several noteworthy findings, including that ACP was associated with lower rates of EOL inpatient care, higher rates of EOL outpatient care, and higher rates of hospice. While the inverse association of EOL outpatient and hospice care contrasting with inpatient care has been noted previously, this study found that the association of ACP with utilization of EOL outpatient care was more pronounced compared with hospice.^41^ As such, this study sought to determine whether the association of ACP on EOL healthcare utilization differed by whether a patient had outpatient care at the EOL (coined “EOL outpatient engagement”) in order to determine whether EOL outcomes observed with billed ACP services simply reflected effects resulting from engagement in outpatient care. This not only suggests that ACP is associated with EOL healthcare utilization irrespective of outpatient engagement, but also suggests that for total EOL expenditure, inpatient care, hospice, SNF, and HHC, the effect of ACP was more pronounced among patients without EOL outpatient engagement. Given that prior studies have found that hospice and avoidance of EOL hospitalizations were associated with increased EOL quality of life, these findings suggest that EOL utilization trends associated with billed ACP may act as a surrogate for better quality of life in the last 30 days of life.

Understanding the mechanisms of how billed ACP reduces overall EOL healthcare expenditures while increasing EOL outpatient expenditures fell outside our study scope. Minimum time requirements stipulated by CMS for billed ACP services may help ensure uniformity in ACP communicative “dosing” to individuals. Future research describing the spectrum of communicative content within billed ACP can build knowledge about how clinicians approach billable ACP encounters. The availability of ACP-focused natural language processing technologies and established learning health systems networks (such as the National Patient-Centered Clinical Research Network) can promote the feasibility of efficient, large-scale evaluation of clinical documentation associated with billed ACP encounters.^42,43^ Research evaluating the comparative effectiveness of billed ACP employing established universal approaches (such as Serious Illness Communication and ACP electronic templates), HF-specific approaches, and unstructured approaches can inform best practices and guide institutional strategies directed at increasing ACP delivery to persons with HF.^44,45^

The results of this study should be interpreted considering multiple limitations common with administrative claims research. The most relevant to this study include (1) findings may be confounded due to latent clinical factors such as noncoded or miscoded diagnoses; (2) a lack of clinically nuanced details that are not reflected in ICD-9/10 billing codes; (3) findings may be confounded with clinic practice/network variation in resources, policies, or other latent factors; (4) unmeasured variation in clinician preferences for end-of-life discussion with patients; and (5) unmeasured patient preferences for end-of-life care. Additionally, it is unknown whether the impact reported in this study results from the minimum requirements of ACP billing or whether ACP billing serves as a proxy for goals communication competencies of clinicians using ACP billing codes. Although CMS claims data offer a comprehensive, national view at ACP utilization for Medicare patients, the scope of claims data does not allow for understanding of the ACP processes and communication content driving our findings, limiting the ability to translate specific evidence-based ACP strategies to clinical settings. While our data set did not lend itself to quality-of-life evaluation, our approach focused on evaluating inpatient and outpatient healthcare utilization sought to identify the impact of billed ACP on promoting time patients can spend in their homes and communities near the EOL.

## CONCLUSION

Billed ACP delivery to individuals with HF was associated with increased EOL outpatient healthcare use, including HHC and hospice service, while being associated with lower rates of inpatient hospital and SNF utilization. These utilization trends result in an overall decrease in EOL healthcare expenditures. As the minority of patients with HF nearing the EOL receive billed ACP services, strategies directed at increasing ACP delivery to this population, especially among patients facing inequities in ACP delivery, may result in shifting HF EOL support services from inpatient to outpatient settings and increase hospice use. Payment policies designed to prioritize ACP delivery in patients with HF may play a pivotal role in changing behaviors and workflows that further incentivize ACP delivery. Comparative effectiveness research focused on identifying billed ACP best practices to improve delivery rates and quality can help inform evidence-based strategies for clinical translation. Identifying EOL healthcare utilization outcomes associated with billed ACP in other disease states can guide policies and focus resources toward individuals who will most benefit at the EOL. Moreover, future studies should consider primary data collection of provider and practice factors that would increase precision in estimates of ACP utilization and identify possible mechanisms for increasing ACP utilization by detangling associations between ACP utilization and other forms of EOL care.

## Supplementary Information

Below is the link to the electronic supplementary material.

Supplementary file1 (DOCX 32 KB)
