# Supplementary material for: Advance Care Planning (ACP) in Medicare Beneficiaries with Heart Failure
Source: J Gen Intern Med. 2024 May 20;39(13):2487–95. doi: 10.1007/s11606-024-08604-1 (PMC11436682; doi:10.1007/s11606-024-08604-1)
Supplement: Supplementary file 1 — Supplementary file1 (DOCX 32 KB) [file 11606_2024_8604_MOESM1_ESM.docx]

Supplementary Online Content

eTable 1: International Classification of Diseases 10th Revision Clinical Modification (ICD-10-CM) diagnosis codes for heart failure

| **Description** | **ICD-10-CM Codes** |
| --- | --- |
| Heart Failure | 425, 428, I43, I50  39891, 40201, 40211, 40291, 40401, 40403, 40411, 40413, 40491, 40493, I099, I110, I130, I132, I255, I420, I425, I426, I427, I428, I429, P290 |

eTable 2: Among patients *with* end-of-life outpatient engagement (i.e., had at least one outpatient encounter in the last 30 days of life; n = 23,394 (48.3%)), descriptive statistics are presented as median (interquartile range) and frequency (relative frequency: %) for patients who did and did not have an ACP encounter.

|  | Total | No ACP N = 20,345 | ACP N = 3,049 |
| --- | --- | --- | --- |
| Number of ACP Encounters |  |  |  |
| 1 | 2,774 (91.0%) | - | 2,774 (91.0%) |
| 2 | 235 (7.7%) | - | 235 (7.7%) |
| 3 | 40 (1.3%) | - | 40 (1.3%) |
| Age | 82 (75, 88) | 82 (75, 88) | 83 (75, 89) |
| Charlson Comorbidity Index | 4 (2, 5) | 4 (2, 5) | 4 (2, 5) |
| Female | 11,778 (50.3%) | 10,208 (50.2%) | 1,570 (51.5%) |
| Race |  |  |  |
| White | 20,623 (88.2%) | 17,935 (88.2%) | 2,688 (88.2%) |
| Black | 1,732 (7.4%) | 1,506 (7.4%) | 226 (7.4%) |
| Other | 1,039 (4.4%) | 904 (4.4%) | 135 (4.4%) |
| Region |  |  |  |
| Midwest | 4,286 (18.3%) | 3,749 (18.4%) | 537 (17.6%) |
| Northeast | 6,115 (26.1%) | 5,403 (26.6%) | 712 (23.4%) |
| South | 7,317 (31.3%) | 6,275 (30.8%) | 1,042 (34.2%) |
| West | 5,676 (24.3%) | 4,918 (24.2%) | 758 (24.9%) |
| Year of Death |  |  |  |
| 2016 | 835 (3.6%) | 732 (3.6%) | 103 (3.4%) |
| 2017 | 2,592 (11.1%) | 2,276 (11.2%) | 316 (10.4%) |
| 2018 | 4,836 (20.7%) | 4,213 (20.7%) | 623 (20.4%) |
| 2019 | 6,962 (29.8%) | 6,072 (29.8%) | 890 (29.2%) |
| 2020 | 8,169 (34.9%) | 7,052 (34.7%) | 1,117 (36.6%) |
|  | 30-day Outcomes | | |
| Total Expenditure ($) | 13,918 (5,937, 25,825) | 14,346 (6,272, 26,427) | 11,171 (4,366, 21,716) |
| Outpatient |  |  |  |
| # of Encounter | 2 (1, 3) | 2 (1, 3) | 3 (1, 5) |
| Total Expenditure ($) | 703 (194, 1,904) | 716 (200, 1,931) | 608 (172, 1,739) |
| Inpatient |  |  |  |
| Any Utilization | 15,126 (64.7%) | 13,444 (66.1%) | 1,682 (55.2%) |
| # of Admissions | 0 (0, 1) | 0 (0, 1) | 0 (0, 1) |
| Total Length of Stay (days) | 3 (0, 8) | 3 (0, 9) | 1 (0, 6) |
| Total Expenditure ($) | 8,952 (0, 18,152) | 9,345 (0, 18,798) | 5,792 (0, 13,863) |
| Hospice |  |  |  |
| Any Utilization | 9,823 (42%) | 8,352 (41.1%) | 1,471 (48.2%) |
| Total Length of Stay (days) | 0 (0, 5) | 0 (0, 4) | 0 (0, 8) |
| Total Expenditure ($) | 0 (0, 1,644) | 0 (0, 1,575) | 0 (0, 1,993) |
| Skilled Nursing Facility |  |  |  |
| Any Utilization | 5,428 (23.2%) | 4,712 (23.2%) | 716 (23.5%) |
| Total Length of Stay (days) | 0 (0, 0) | 0 (0, 0) | 0 (0, 0) |
| Total Expenditure ($) | 0 (0, 0) | 0 (0, 0) | 0 (0, 0) |
| Home Healthcare |  |  |  |
| Any Utilization | 4,184 (17.9%) | 3,654 (18%) | 530 (17.4%) |
| Total Length of Stay (days) | 0 (0, 0) | 0 (0, 0) | 0 (0, 0) |
| Total Expenditure ($) | 0 (0, 0) | 0 (0, 0) | 0 (0, 0) |

eTable 3: Among patients *without* end-of-life outpatient engagement (i.e., had at least one outpatient encounter in the last 30 days of life; n = 25,072 (51.7%), descriptive statistics are presented as median (interquartile range) and frequency (relative frequency: %) for patients who did and did not have an ACP encounter.

|  | Total | No ACP N = 23,715 | ACP N = 4,406 |
| --- | --- | --- | --- |
| Number of ACP Encounters |  |  |  |
| 1 | 1,244 (91.7%) | - | 1,244 (91.7%) |
| 2 | 98 (7.2%) | - | 98 (7.2%) |
| 3 | 15 (1.1%) | - | 15 (1.1%) |
| Age | 84 (77, 90) | 84 (77, 90) | 83 (77, 90) |
| Charlson Comorbidity Index | 4 (2, 5) | 4 (2, 5) | 4 (2, 5) |
| Female | 13,060 (52.1%) | 12,372 (52.2%) | 688 (50.7%) |
| Race |  |  |  |
| White | 22,057 (88%) | 20,865 (88.0%) | 1,192 (87.8%) |
| Black | 1,854 (7.4%) | 1,754 (7.4%) | 100 (7.4%) |
| Other | 1,161 (4.6%) | 1,096 (4.6%) | 65 (4.8%) |
| Region |  |  |  |
| Midwest | 3,612 (14.4%) | 3,431 (14.5%) | 181 (13.3%) |
| Northeast | 6,172 (24.6%) | 5,767 (24.3%) | 405 (29.8%) |
| South | 8,061 (32.2%) | 7,705 (32.5%) | 356 (26.2%) |
| West | 7,227 (28.8%) | 6,812 (28.7%) | 415 (30.6%) |
| Year of Death |  |  |  |
| 2016 | 738 (2.9%) | 698 (2.9%) | 40 (2.9%) |
| 2017 | 2,787 (11.1%) | 2,614 (11.0%) | 173 (12.7%) |
| 2018 | 5,262 (21.0%) | 4,967 (20.9%) | 295 (21.7%) |
| 2019 | 7,239 (28.9%) | 6,838 (28.8%) | 401 (29.6%) |
| 2020 | 9,046 (36.1%) | 8,598 (36.3%) | 448 (33.0%) |
|  | 30-day Outcomes | | |
| Total Expenditure ($) | 9,940 (5,660, 21,204) | 10,084 (5,613, 21,656) | 8,601 (6,171, 13,801) |
| Inpatient |  |  |  |
| Any Utilization | 15,126 (64.7%) | 13,444 (66.1%) | 1,682 (55.2%) |
| # of Admissions | 0 (0, 1) | 1 (0, 1) | 0 (0, 1) |
| Total Length of Stay (days) | 0 (0, 8) | 0 (0, 8) | 0 (0, 4) |
| Total Expenditure ($) | 0 (0, 15054) | 0 (0, 15,387) | 0 (0, 9,819) |
| Hospice |  |  |  |
| Any Utilization | 14,445 (57.6%) | 13,448 (56.7%) | 997 (73.5%) |
| Total Length of Stay (days) | 3 (0, 32) | 3 (0, 31) | 25 (0, 41) |
| Total Expenditure ($) | 1,325 (0, 5,960) | 1,170 (0, 5,776) | 4,941 (0, 7,603) |
| Skilled Nursing Facility |  |  |  |
| Any Utilization | 4,066 (16.2%) | 3,923 (16.5%) | 143 (10.5%) |
| Total Length of Stay (days) | 0 (0, 0) | 0 (0, 0) | 0 (0, 0) |
| Total Expenditure ($) | 0 (0, 0) | 0 (0, 0) | 0 (0, 0) |
| Home Healthcare |  |  |  |
| Any Utilization | 2,623 (10.5%) | 2,507 (10.6%) | 116 (8.5%) |
| Total Length of Stay (days) | 0 (0, 0) | 0 (0, 0) | 0 (0, 0) |
| Total Expenditure ($) | 0 (0, 0) | 0 (0, 0) | 0 (0, 0) |

eTable 4: Multivariable analysis results are presented as incidence rate ratio (IRR) or odds ratio (OR) as well as the corresponding 95% confidence intervals for all utilization outcomes, comparing ACP encounter with no ACP encounter, stratified by end-of-life outpatient engagement.

|  | With EOL Outpatient Engagement | | | Without EOL Outpatient Engagement | | |
| --- | --- | --- | --- | --- | --- | --- |
|  | *IRR/^£^OR | 95% CI | p | *IRR/^£^OR | 95% CI | p |
| Total Expenditure ($) | *0.81 | 0.78-0.85 | <0.001 | *0.75 | 0.69-0.82 | <0.001 |
| Outpatient |  |  |  |  |  |  |
| # of Encounter | *1.53 | 1.49-1.57 | <0.001 | ^-^ | ^-^ | ^-^ |
| Total Expenditure ($) | *0.88 | 0.84-0.92 | <0.001 | ^-^ | ^-^ | ^-^ |
| Inpatient |  |  |  |  |  |  |
| Any Utilization | ^£^0.62 | 0.57-0.68 | <0.001 | ^£^0.44 | 0.38-0.49 | <0.001 |
| # of Admissions | *0.83 | 0.80-0.87 | <0.001 | *0.84 | 0.81-0.87 | <0.001 |
| Total Length of Stay (days) | *0.72 | 0.68-0.77 | <0.001 | *0.59 | 0.53-0.66 | <0.001 |
| Total Expenditure ($) | *0.72 | 0.67-0.78 | <0.001 | *0.55 | 0.48-0.63 | <0.001 |
| Hospice |  |  |  |  |  |  |
| Any Utilization | ^£^1.36 | 1.25-1.47 | <0.001 | ^£^2.32 | 2.04-2.63 | <0.001 |
| Total Length of Stay (days) | *1.36 | 1.24-1.50 | <0.001 | *1.79 | 1.60-2.00 | <0.001 |
| Total Expenditure ($) | *1.24 | 1.14-1.35 | <0.001 | *1.64 | 1.46-1.85 | <0.001 |
| Skilled Nursing Facility |  |  |  |  |  |  |
| Any Utilization | ^£^1.10 | 1.01-1.21 | 0.025 | ^£^0.60 | 0.50-0.71 | <0.001 |
| Total Length of Stay (days) | *1.14 | 0.99-1.32 | 0.08 | *0.60 | 0.47-0.78 | <0.001 |
| Total Expenditure ($) | *1.14 | 1.03-1.27 | 0.011 | *0.66 | 0.57-0.77 | <0.001 |
| Home Healthcare |  |  |  |  |  |  |
| Any Utilization | ^£^0.96 | 0.87-1.07 | 0.49 | ^£^0.77 | 0.63-0.94 | 0.011 |
| Total Length of Stay (days) | *0.88 | 0.75-1.03 | 0.11 | *0.94 | 0.69-1.29 | 0.71 |
| Total Expenditure ($) | *0.77 | 0.70-0.84 | <0.001 | *1.01 | 0.88-1.15 | 0.93 |
